# Clinical strategies for ART treatment of infertile women with advanced maternal age

**DOI:** 10.1002/rmb2.12240

**Published:** 2018-11-14

**Authors:** Koji Nakagawa, Keiji Kuroda, Rikikazu Sugiyama

**Affiliations:** ^1^ Center for Reproductive Medicine and Implantation Research Sugiyama Clinic Shinjuku Tokyo Japan

**Keywords:** advanced maternal age, conventional insemination, double OPU, ICSI, ovarian stimulation

## Abstract

**Background:**

An ever‐increasing number of women in our country with advanced maternal age are choosing to achieve pregnancy. This means effective strategies are needed for infertile patients. Questions arise, however, concerning the need for ovarian stimulation, and, if so, whether intracytoplasmic sperm injection (ICSI) is better than conventional insemination for those women who may have only one mature oocyte.

**Methods:**

We evaluated our data to answer these questions. Herein, we also introduce our strategy for patients who show unsynchronized follicular growth.

**Main findings:**

Ovarian stimulation in ART treatment for patients with advanced maternal age has resulted in the achievement of higher pregnancy rates, and therefore, this form of stimulation is often selected. Based on our data, ICSI as an insemination procedure has not improved clinical pregnancy rates compared with conventional insemination and has actually decreased the clinical pregnancy rates.

**Conclusion:**

In this article, we reviewed and compared the protocols and strategies that are available to increase the number of developed embryos for the patients with advanced maternal age. We hope that this review will be helpful for both patients and clinicians.

## 1 INTRODUCTION

There were approximately 1.43 million newborns in Japan in 1985, which is 25% higher compared with the number of births in 2015 (1.05 million). In 1985, however, only 0.6% of the newborns were derived from the women who were more than 40 years of age, while the rate in 2015 was surprisingly increased (22.8%).^1^, ^2^ There were 8224 newborns delivered to women who were between 40 and 44 years of age in 1985, and this number increased to 52 577 in 2015—approximately 5% of the total number of births for that year (Table 1).^1^, ^2^ Delivery by women with advanced maternal age used to be rare, but it is no longer uncommon. The reasons for the increase compared with 30 years ago seem to be 2‐fold: a trend toward later marriages and the progress of the infertility treatment.

**Table 1:** The number of newborns every 5 y between 1985 and 2015 in Japan^a^^,^ b

Age, years	1985	1995	2005	2015
Total	1 431 577	1 187 064	1 062 530	1 005 656
25‐29	682 885	494 717	339 328	622 512
30‐34	381 466	371 733	404 700	364 863
35‐39	93 501	100 053	153 440	228 289
40‐44	8224	12 472	19 750	52 577
45	245	414	598	1308

a Summary of vital statistics (number, rates interval of occurrence), 2015.

b Summary of vital statistics (number, rates interval of occurrence), 1985.

The first successful in vitro fertilization (IVF) treatment in Japan was reported in 1983,^3^ but this treatment was uncommon for the infertility treatment in that era due to its specificity; therefore, this treatment did not add to the official birthrate. IVF treatment has now become common for the patients who wish to have a baby. Thirty years ago, the infertile patients who were between 40 and 44 years of age found it quite difficult to conceive naturally or even via the use of intrauterine insemination. ART and IVF treatments now play an important role in the field of infertility treatment and have made a significant contribution to the increased number of newborns. The number of infertile patients with advanced maternal age (≥40 years) has seen a recent increase. Even with the increased use of ART treatment, however, the pregnancy rates are not yet satisfactory among patients who are more than 40 years of age. The miscarriage rate, unfortunately, has increased due to these increases in the advanced maternal age.^4^ Indeed, there were 6658 cycles planned for oocyte pickup (OPU) for ART treatment at Sugiyama Clinic between January and December in 2016, and 48.1% (3201 cycles) of them were for patients who were more than 40 years of age. This recent trend is not exclusive to our clinic, but in fact is becoming common in Japan.

The clinicians working at the Department of Reproductive Medicine and in clinics that specialize in infertility treatment encounter many patients among these age‐groups every day. Even though the success rate of IVF treatment for these patients is relatively low even when using ART treatment, these may be the only viable options for infertile patients with advanced maternal age. When devising strategies to overcome the poor success rate, two main questions often must be answered: one is that whether ovarian stimulation is needed or not, and the other is whether intracytoplasmic sperm injection (ICSI) be better than conventional insemination as an insemination method for patients who may have only one mature oocyte? In this article, we expanded on and discuss these two main points. In addition, our strategy for the patients showing unsynchronized follicular growth is also introduced.

## 2 IS OVARIAN STIMULATION NEEDED FOR THE PATIENTS WITH ADVANCED MATERNAL AGE?

Between 2012 and 2016, a total of 3545 cycles were recruited for analysis. The patients recruited in these treatment cycles satisfied the following two conditions: They were more than 40 years of age at the time of OPU and they had used OPU <2 times. Moreover, all patients had received fresh embryo transfers, and patients receiving cryopreserved embryos were excluded, because these embryos did not always reflect the maternal age (cryopreservation of embryos could make the difference of the patients’ age between the cryopreservation day and embryo transfer day). All cycles were divided into one of two groups: the stimulation group and the minimal stimulation group. There were 1666 cycles in the stimulation group, and this group received ovarian stimulation that included either the gonadotropin‐releasing hormone (GnRH)‐agonist long protocol^5^ or the GnRH‐agonist short protocol,^6^ along with either clomiphene citrate (CC) only or a combination of CC and recombinant follicle‐stimulating hormone (FSH).^7^ There were 1879 cycles in the minimal stimulation group, which used only CC or letrozole, or a natural ovulatory cycle. To calculate and evaluate the clinical pregnancy rate, OPU cycles were used rather than embryo transfer cycles. Patients and clinicians alike usually act as an index to evaluate the effectiveness of ART. However, this rate only indicates the number of patients who were able to achieve pregnancy by receiving embryo transfer, which does not reflect the number receiving OPU. This rate does not include the number of patients who received OPU without an embryo for embryo transfer. Patients often inquire about the success rate for OPU. Therefore, the pregnancy rate per OPU cycle was selected to evaluation. Clinical pregnancy was defined as confirmation via vaginal ultrasound of the existence of a gestational sac in the uterine cavity.

**Figure 1:**
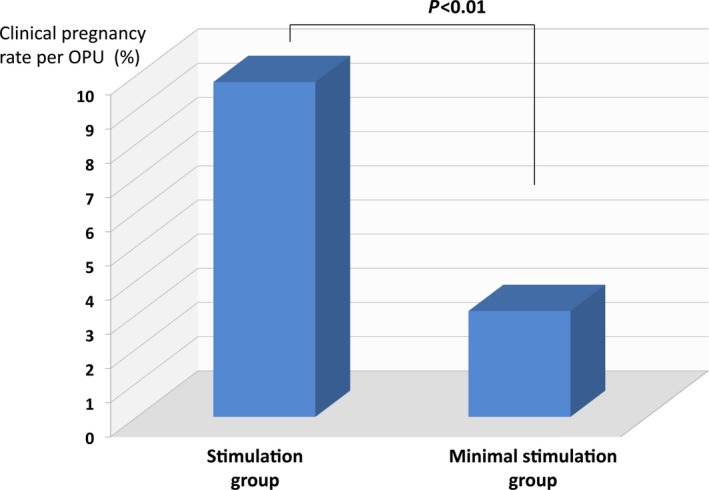
This graph indicates the clinical pregnancy rates per oocyte pickup in the stimulation and minimal stimulation groups. The rate in the stimulation group was 9.8% and was significantly higher than that in the minimal stimulation group (3.1%, *P* < 0.01)

The ART outcomes for the two groups are listed in Table 2. The number of cycles scheduled to receive OPU in the stimulation and minimal stimulation groups was 1666 and 1879, respectively. For patients in the stimulation group, 7.1% (n = 118) of total cycles resulted in no usable oocytes for the following reasons: cancelation of OPU for just after ovulation (n = 48), no oocyte to retrieve (n = 26), or retrieval of a degenerated oocyte (n = 44). By contrast, the percentage of patients who could obtain no usable oocytes in the minimal stimulation group was 49.0% (n = 920), which was significantly higher than that in the stimulation group (*P* < 0.01). The reasons were the same: n = 580, n = 142, and n = 198, respectively. In the stimulation group, the OPU cancelation rate was 2.8%, which was significantly lower than that in the minimal stimulation group (30.9%, *P* < 0.01). In the stimulation group, patients received some agents to prevent ovulation such as GnRH‐agonist, GnRH‐antagonist, or CC,^8^ and this was one of the reasons for the lower cancelation rate in the stimulation group. The use of GnRH‐agonist and GnRH‐antagonist during ovarian stimulation could suppress LH secretion from the pituitary gland and consequently could prevent ovulation; by contrast, CC acts antagonistically to the estradiol receptor at the hypothalamus level, inhibiting both negative and positive feedbacks, and resulting in the suppression of ovulation during ovarian stimulation.^8^ The average number of retrieved oocytes in the stimulation group was 4.2 ± 1.8, which was significantly higher than that in the minimal stimulation group (1.2 ± 0.3, *P* < 0.01). In the stimulation group, 1285 cycles of fresh embryo transfers were performed, and 164 patients achieved pregnancy for a clinical pregnancy rate per OPU of 9.8%. On the other hand, in the minimal stimulation group, at 543 cycles fresh ET was performed, and 59 pregnancies were achieved. The clinical pregnancy rate per OPU was 3.1%, which was significantly lower than for the stimulation group (Figure 1; *P* < 0.01). It was considered that two reasons might cause this result. One was that a higher number of retrieved oocytes were collected in the stimulation group compared with that in the minimal stimulation group. The other was that the rate of unusable oocytes in the stimulation group was lower compared with that in the minimal stimulation group. The half of the cycles scheduled of oocyte retrieval in the minimal stimulation group could not proceed to the further step to fertilize. As a consequence, ovarian stimulation for the patients with advanced maternal age in ART treatment resulted in the achievement of a higher pregnancy rate; therefore, ovarian stimulation is recommended for these types of patients who receive ART treatment.

**Table 2:** Clinical outcomes in the stimulation and minimal stimulation groups

	Stimulation group	Minimal stimulation group
Number of cycles scheduled to receive OPU	1666	1879
Number of cycles resulted in no usable oocytes, n (%)	118 (7.1)	920 (49.0)*
Cancelation of OPU, n (%)	48 (2.9)	580 (30.9)*
Retrieval of degenerated oocyte, n (%)	44 (2.6)	142 (7.6)*
No oocyte to retrieve, n (%)	26 (1.6)	198 (10.5)*
Number of retrieved oocytes (mean ± SD)	4.2 ± 1.8	1.2 ± 0.3*
Number of fresh embryo transfer cycles, n	1.285	543
Number of clinical pregnancy, n	164	59
Clinical pregnancy rate per OPU, %	9.8	3.1

OPU, oocyte pick‐up.

vs stimulation group, *P* < 0.01.

The only nearly 10% of the patients in the stimulation group could not obtain usable oocytes in spite of receiving ovarian stimulation, but the physicians with reproduction should suggest further plans or protocols for them. The further plans were selected according to the AMH values or basal FSH level of the patients. When the patients showed low AMH value (<1.0 ng/mL) or elevated basal FSH level (≥15 IU/L), we usually recommend them to change past protocol to minimal ovarian stimulation protocol using only CC or letrozole for their further stimulation. Because these patients with diminished ovarian reserve will not be expected to get more numbers of the oocytes even though strong stimulation using GnRH‐agonist long protocol, short protocol or GnRH‐antagonist protocol, except only increasing their economical and physical burden. By contrast, when the patients showed moderate AMH value (≥1.0 ng/mL) or normal basal FSH (<15 IU/L) who showed inadequate ovarian response stimulated by the conventional stimulation protocol, we will select same ovarian protocol for their next treatment.

## 3 IS ICSI NEEDED FOR THE PATIENTS WITH ADVANCED MATERNAL AGE WHO CAN OBTAIN ONLY ONE OOCYTE?

Clinicians sometimes face a situation whereby patients have only one follicle on the day of maturation trigger with or without ovarian stimulation, particularly those with advanced maternal age. Within the time period and recruited cycles mentioned above, the number of cycles that showed only one follicle for oocyte retrieval in the stimulation and minimal stimulation groups was 338 and 1791, respectively. In the stimulation group, at 107 cycles embryo transfer was performed, and six pregnancies were achieved. The clinical pregnancy rate per OPU and ET was 1.9% and 17.8%, respectively. By contrast, in the minimal stimulation group, 39 pregnancies by the 385 transfers were achieved for clinical pregnancy rates per OPU and ET of 2.3% and 10.1%, respectively (Table 3). There were no significant differences between the two groups. Based on these data, ovarian stimulation would not have increased the clinical pregnancy rate for patients who showed only one developed follicle in reaction to the ovarian stimulation.

**Table 3:** Clinical outcomes of the cycles showing only one follicle in the stimulation and minimal stimulation groups

	Stimulation group	Minimal stimulation group
Number of cycles showing only one follicle	338	1791
Number of embryo transfer cycles	107	543
Number of freeze‐all cycles	26	122
Number of clinical pregnancies	6	39
Clinical pregnancy rate per oocyte pickup^a^, %	1.9	2.3
Clinical pregnancy rate per embryo transfer, %	17.8	10.1

*vs stimulation group, *P* < 0.01.

a Excluding the freeze‐all cycles.

**Figure 2:**
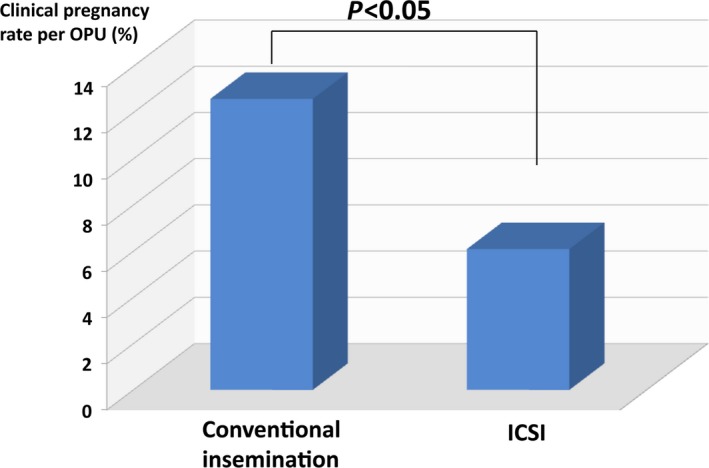
The clinical pregnancy rate per ET derived from the conventional insemination was 12.6%, which was significantly higher than that derived from intracytoplasmic sperm injection (5.2%, *P* < 0.05)

When clinicians must choose an insemination procedure for the patients with only one oocyte, the option is either ICSI or conventional insemination. Based on Japanese ART data, the ICSI cycle is more involved than a conventional insemination cycle, and this difference has grown for each of the past 3 years.^4^ This trend seems to be a worldwide phenomenon. In general, the fertilization rate of ICSI is thought to be similar to that of conventional insemination.^9^, ^10^ However, many patients and clinicians fervently believe that ICSI might be superior to conventional insemination with regard to fertilization, because ICSI directly injects spermatozoa to the oocyte. For this reason, it is not uncommon that ICSI is selected as the insemination procedure for the patients who have only one oocyte. Recently, we reported that the insemination procedure (conventional insemination or ICSI) had no influence on the outcome of ART treatment, and collecting only one oocyte is not an indication for ICSI.^11^ Therefore, for the patients who could obtain only one oocyte, we compared the clinical pregnancy rate by ICSI‐derived embryo with that by conventional insemination‐derived embryo to evaluate which was more productive. The clinical pregnancy rate per ET derived from conventional insemination was 12.6%, which was significantly higher than that derived from ICSI (5.2%, *P* < 0.05; Figure 2). Based on our data, compared with conventional insemination, ICSI did not improve the clinical pregnancy rate.

## 4 DOUBLE OOCYTE PICKUP FOR THE PATIENTS WITH DOR AND SHOWING UNSYNCHRONIZED FOLLICULAR GROWTH DURING ART TREATMENT

For infertile patients with diminished ovarian reserve (DOR), particularly lower anti‐Müllerian hormone (AMH) levels (<1.0 ng/mL), or an antral follicle count (AFC) of 1‐2 during the menstrual period, clinicians often struggle with the selection of an ovarian stimulation protocol, because the number of retrieved oocytes is a critical point for achieving pregnancy. Ovarian stimulation with high‐dose gonadotropins, however, sometimes only increases a patient's burden and does not contribute to the number of grown follicles and retrieved oocytes. In order to achieve pregnancy, ART treatment can provide many oocytes with one OPU procedure, which could improve the chances of selecting the best embryo or blastocyst for embryo transfer. It is difficult, however, for DOR patients to obtain a plurality of oocytes in one OPU. On the other hand, clinicians sometimes observe unsynchronized follicular growth during ovarian stimulation. In those cases, the dominant follicle size is a crucial maturation trigger, and subsequently, inadequately developed follicles are removed as a target of OPU, despite their value to DOR patients.

Previously during ART treatment, if a patient's serum progesterone (P4) concentration was elevated (≥1.5 ng/mL) on the day maturation was triggered, OPU was usually canceled. This was the policy because an elevation of P4 reduces the rate of implantation ,^12^, ^13^ which could affect oocyte quality.^14^ In a recent effort to avoid fresh embryo transfer, we reported that P4 elevation (≥1.5 ng/mL) on the day of maturation trigger could not decrease the pregnancy rate.^15^ In the present study, moreover, we found that P4 elevation affected neither oocyte recovery rate nor embryo development rate.^15^ On the other hand, the effectiveness of randomly beginning a protocol of ovarian stimulation has been reported.^16^, ^17^ This protocol indicated that the decision to begin ovarian stimulation should not be related to the menstrual cycle.

When patients with DOR and low AMH levels receive ovarian stimulation, clinicians sometimes struggle with unsynchronized follicular growth. In these cases, OPU is performed according to the dominant follicle, and a small one could be ignored, or immature oocytes could be retrieved from the dominant follicle; as a consequence, this procedure would not improve the ART outcome. Therefore, for patients with DOR, a small follicle could be quite important and valuable. For these patients, we typically perform a double OPU. In this procedure, OPU is performed twice in one treatment cycle. The first OPU is performed for only the dominant follicle following the maturation trigger; continuous ovarian stimulation is performed for the second and smaller follicle, and a second OPU is performed after the maturation trigger for the second follicle. The efficacy of double OPU was analyzed for DOR patients ≥40 years of age who showed unsynchronized follicular growth, and the outcome of DOR was analyzed.

From April 2016 to October 2017, we recruited patients with low AMH level (<1.0 ng/mL) who were more than 40 years of age and had received ovarian stimulation using only CC or a combination of CC and recombinant follicle‐stimulating hormone (r‐FSH), and 13 patients met the qualifications. The average age of the patients was 41.6 years, and the mean AMH (ng/mL) and basal follicle‐stimulating hormone (FSH) levels (IU/L) were 0.47 and 15.1, respectively. Ten of the 13 patients were administrated either CC or CC with r‐FSH injection.

## 5 A PROTOCOL OF DOUBLE OPU AND THE OUTCOME OF THIS PROCEDURE

**Figure 3:**
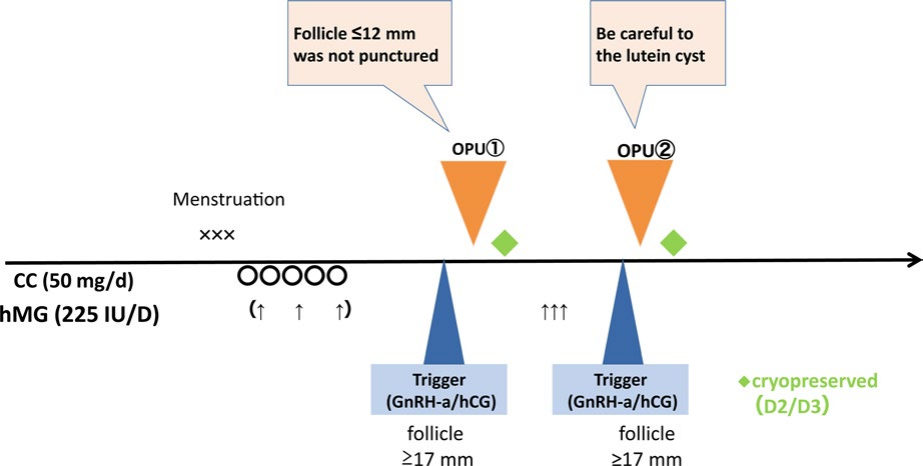
The protocol for double oocyte pickup (OPU) is shown based on our mild stimulation protocol with CC and recombinant‐follicle‐stimulating hormone (r‐FSH)^7^ and is briefly described as follows. Patients received 50 mg of CC (Clomid^®^; Merck) daily between D3 and D7 of the menstrual cycle, with or without 225 IU of r‐FSH (Gonal‐F^®^; Merck) injection. The leading follicle should reach 17 mm in diameter, following a maturation trigger with either 5000 IU of human chorionic gonadotropin (hCG; HCG 5000F^®^; Fuji Pharma) or 300 μg of gonadotropin‐releasing hormone (GnRH)‐agonist (Buserequr^®^; Fuji Pharma) performed 35 h before OPU. During OPU, follicles 12 mm or less in diameter were not punctured. On the day of OPU, ovarian stimulation with r‐FSH injection was started to induce growth in the smaller follicles. When the follicles reached 17 mm in diameter, the maturation trigger was completed, and 36 h later, a second OPU was performed. The embryos derived from the first and second OPU were cryopreserved

The protocol for double OPU is shown in Figure 3 based on our mild stimulation protocol with CC and r‐FSH^7^ and is briefly described as follows. Patients received 50 mg of CC (Clomid^®^; Merck, Tokyo, Japan) daily between D3 and D7 of the menstrual cycle either with or without an injection of 225 IU of r‐FSH (Gonal‐F^®^; Merck). The leading follicles reached 17 mm in diameter with a maturation trigger of either 5000 IU of human chorionic gonadotropin (hCG; HCG 5000F^®^; Fuji Pharma, Toyama, Japan) or 300 μg of gonadotropin‐releasing hormone (GnRH)‐agonist (Buserequr^®^; Fuji Pharma) performed 35 hours before OPU. During OPU, follicles 12 mm or less in diameter were not punctured. On the day of OPU, ovarian stimulation with r‐FSH injection was started to promote the growth of smaller follicles. These follicles were expected to reach 17 mm in diameter with a maturation trigger, and a second OPU was scheduled for 36 hours later. The embryos derived from the first and second OPU were cryopreserved. Seven out of the 13 patients received hCG as a maturation trigger at the first OPU. After the first OPU, nine of the patients received r‐FSH, and nine of the 13 were administrated hCG as a second maturation trigger. The progesterone concentration on the day of the second maturation trigger was 15.6.ng/mL. The outcomes of the double OPU for the DOR patients showing unsynchronized follicular growth are shown in Table 4. The first and second OPUs resulted in the retrieval of 1.3 and 1.2 oocytes, respectively, for a total of 2.5. The mean number of MII oocytes on the first and second OPU was 1.1 and 1.2, respectively, and MII rates on the first and second OPU were 62.5% and 100%, respectively. The mean number of fertilized oocytes on the first and second OPU was 1.1 and 0.9, respectively, and the percentage of morphologically good‐quality (MGQ) embryos on the first and second OPU was 33.3% and 25.0%, respectively. Embryos were defined as MGQ embryos when they had developed to at least 6‐cell stage with <20% fragmentation.^18^ This system of embryo assessment was based on the classification system described by Veeck.^19^ All embryos were not tried to develop to the blastocyst stage due to their small number of retrieved and fertilized oocytes.

**Table 4:** Background and ART outcomes of the patients who received double oocyte pickup (OPU)

Patients, n	13
Age, years^a^	41.6
AMH values, ng/mL^a^	0.47
Basal follicle‐stimulating hormone values, IU/L^a^	15.1
Ovarian stimulation: natural/CC/CC + hMG, n	3/5/5
First maturation trigger: hCG/GnRH‐a, n	7/6
Ovarian stimulation after the first OPU: hMG/CC, n	9/4
Second maturation trigger: hCG/GnRH‐a, n	9/4
Progesterone concentration on the day of the second maturation trigger, ng/ml^a^	15.6
Number of retrieved oocytes on the first OPU, n^a^	1.3
Number of MII oocytes on the first OPU, n^a^	1.1
Percentage of MII oocytes on the first OPU, %	62.5
Number of fertilized oocytes on the first OPU, n^a^	1.1
Percentage of MGQ embryos on the first OPU, %	33.3
Number of retrieved oocytes on the second OPU, n^a^	1.2
Number of MII oocytes on the second OPU, n^a^	1.2
Percentage of the MII oocytes on the second OPU, %	100
Number of fertilized oocytes on the second OPU, n^a^	0.9
Percentage of MGQ embryos on the second OPU, n^a^	25.0
Total number of collected oocytes, n^a^	2.5
Number of patients who acquired cryopreserved embryos, n	12

AMH, anti‐Müllerian hormone; CC, clomiphene citrate; hMG, human menopausal gonadotropin; hCG, human chorionic gonadotropin; GnRH‐a, gonadotropin‐releasing hormone‐agonist; MGQ, morphologically good quality.

a Mean.

**Figure 4:**
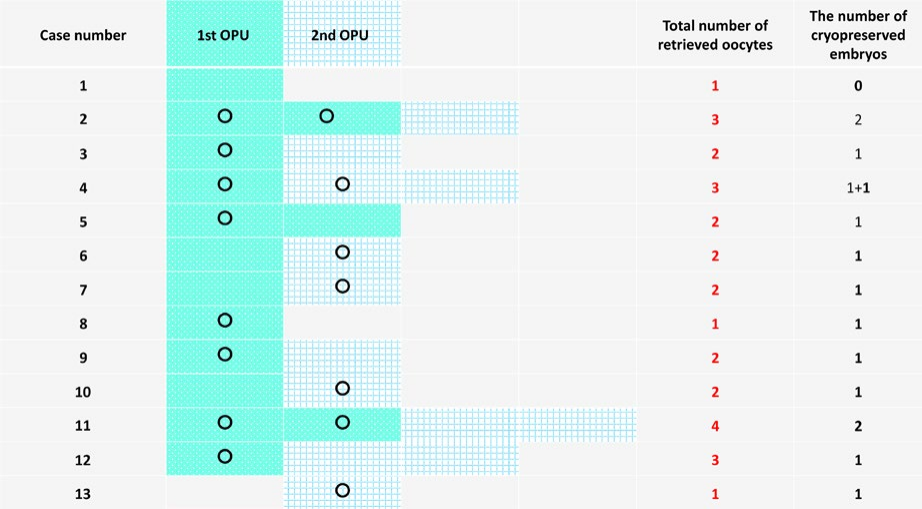
At the first oocyte pickup (OPU), 12 out of 13 patients received oocytes, and 11 out of 13 received oocytes at the second OPU. Four patients (Patients 6, 7, 10, and 13) were able to cryopreserve embryos derived from the second OPU. Among them, a pregnancy was confirmed for Patient 4. 〇: oocytes that could be cryopreserved; 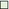: retrieved oocytes on the first OPU; 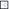: retrieved oocytes on the second OPU

Twelve out of the 13 patients were able to obtain cryopreserved embryos. The OPU results for each patient are shown in Figure 4. At the first OPU, 12 out of the 13 patients received oocytes, and 11 out of the 13 received oocytes at the second OPU. Four patients could get cryopreserved embryos derived from the second OPU, and pregnancy was confirmed for Patient 4 (Figure 4). Although the patients obtained only 1.3 eggs by the conventional procedure, the number was increased to about 2.5 eggs with a double OPU, and about 90% of them received cryopreserved embryos. When the clinicians met unsynchronized follicular growth, they ignored the smaller ones on the first OPU, and the smaller ones were then stimulated with gonadotropins for the second OPU. This double OPU might be an effective procedure among the DOR patients to increase the number of collected oocytes (/patient) from the first and second rounds of OPU. During the first and second rounds of OPU, the usable embryos for cryopreservation numbered 10 and 5, respectively, and as a consequence, 12 of the patients were able to obtain cryopreserved embryos. Of these 12 patients, four were unable to obtain cryopreserved embryos without a second round of OPU.

Progesterone elevation on the day of the maturation trigger neither decreased the pregnancy rate when a fresh embryo transfer was avoided, nor affected the embryo development rate. Furthermore, a random start was reported for ovarian stimulation.^16^, ^17^ This indicated that the start of ovarian stimulation was not related to the menstrual cycle. This random‐start protocol has now been adapted for the patients who immediately face a surgical procedure, chemotherapy, or radiation therapy due to cancer, and ovarian stimulation can be started any time with or without an elevation of progesterone concentration. The double OPU outlined here, however, might be useful for DOR patients who show unsynchronized follicular growth. The smaller follicles (<12 mm in diameter) on the day of maturation trigger are not suitable for OPU, because only immature oocytes are normally retrieved from these smaller follicles. These could develop via reaction by ovarian stimulation using CC with or without r‐FSH administration, although more than 10 ng/mL of progesterone concentration represents a so‐called secretory phase. Moreover, usable embryos could be obtained from a second round of OPU. Based on our data, follicle development and oocyte quality are not dependent on an elevation of progesterone. The only problem would be the increased cost regarding double OPU. In our clinic, a second round of OPU is discounted considerably, which makes it easier for patients to choose a double OPU. More experience is needed for the establishment of a universal double‐OPU protocol, which will require the application of this protocol to more patients.

## 6 CONCLUSIONS

In our country, the number of women with advanced maternal age who choose to become pregnant increases every year. This means effective strategies are needed as soon as possible. There is no doubt that the transfer of euploid embryos or blastocysts is the most effective strategy for these women to achieve pregnancy, but the preimplantation genetic testing for aneuploidy (PGTA) is not allowed in our country. Moreover, the number of retrieved oocytes or developed embryos among the patients with advanced maternal age would likely be insufficient for PGTA due to either poor ovarian response or diminished ovarian reserve. In this article, we reviewed and compared the protocols and strategies that are available to increase the number of developed embryos. We hope that this review will be helpful for both patients and clinicians.

## HUMAN RIGHTS STATEMENTS AND INFORMED CONSENT

We obtained a written informed consent from each participant couple. This study was approved by the ethical committee of Sugiyama Clinic.

## APPROVAL OF ETHICS COMMITTEE

All the procedures were followed in accordance with the ethical standards of the responsible committee of Sugiyama Clinic and with the Helsinki Declaration of 1964 and its later amendments.

## CLINICAL TRIAL REGISTRY

This article was not applicable to clinical trial registry.

## CONFLICT OF INTEREST

The authors have stated explicitly that there are no conflict of interests in connection with this article.
